# Editorial: Cardiac Remodeling: New Insights in Physiological and Pathological Adaptations

**DOI:** 10.3389/fphys.2017.00751

**Published:** 2017-09-26

**Authors:** Leonardo Roever, Antonio C. Palandri Chagas

**Affiliations:** ^1^Federal University of Uberlândia, Uberlândia, Brazil; ^2^Heart Institute (InCor), HCFMUSP University of São Paulo Medical School, São Paulo, Brazil; ^3^Faculty of Medicine ABC, Santo André, Brazil

**Keywords:** cardiac remodeling, physiology, heart failure, fibrosis, inflammation

## Introduction

Cardiac remodeling (CR) is a complex process involving cardiac myocyte growth and death, vascular rarefaction, fibrosis, inflammation, and electrophysiological alterations (Burchfield et al., 2013; Xie et al., 2013). CR can be conceptualized as a set of cellular, cardiac and interstitial changes in the heart, manifested clinically by changes in the size, mass (hypertrophy and atrophy), geometry (wall thickness and heart shape) and function, in response to a given stimulus—it is one of the major responses of the heart to biomechanical stresses and pathological stimuli (Zornoff et al., 2009; Gajarsa and Kloner, 2011; Braunwald, 2013; Heusch et al., 2014; Sekaran et al., 2017). We can also detect the presence of scarred areas, fibrosis and inflammatory infiltrate.

The most common methods used to detect these CR are echocardiography, ventriculography, tomography, magnetic resonance imaging, and most recently PET cardiac scans (Anand et al., 2002; Presotto et al., 2016). Biomarkers have been also used as indicators of CR, among them we can mention myosin heavy chain alterations (decrease of alpha and increase of beta-myosin), increase of caveolin, increase in neuronal nitric oxide synthase expression and increase of angiotensin converting enzyme, increase of alpha-actin, increase of galectin-3, increase of natriuretic peptides, decrease of GLUT-4 and increase Of GLUT-1, decrease of SERCA2a. Another feature is the substitution of the energetic preferential substrate of fatty acids for glucose (Swynghedauw, 2006; Eschalier et al., 2014; Liquori et al., 2014).

The aim of the present research topic was therefore to bring together key experiments, advances and new findings related to several aspects of CR.

Cardiac dysfunction is the main implication of the process of remodeling. It begins with genetic alterations in response to an insult to the heart, which is consequently manifested by cellular and molecular cardiac alterations, which result in progressive loss of ventricular function, initially asymptomatic and later with signs and symptoms characteristic of heart failure (Cohn et al., 2000; Azevedo et al., 2016). These stem from consequence of an excess of stimulation of the sympathetic system and the renin-angiotensin-aldosterone system which stimulates intracellular signaling pathways with a consequent increase in protein synthesis in myocytes and fibroblasts causing cellular hypertrophy and fibrosis, activation of growth factors, activation of metalloproteinases, hemodynamic overload by vasoconstriction and water retention, increase of oxidative stress and direct cytotoxic effect, leading to cell death by necrosis or apoptosis (Florea and Cohn, 2014; Sayer and Bhat, 2014). Another factor that is potentially responsible for altered cardiac function in the remodeling heart is the energy deficit, which results from the imbalance between supply and consumption of oxygen, including also the decrease in use of free fatty acids and increased use of glucose as a consequence of the reduction of β-oxidation, and abnormal accumulation of triglycerides and lipotoxicity may occur (Doenst et al., 2013; Santos et al., 2014; Nishida and Otsu, 2016).

Autophagy is an intracellular process in which defective or unnecessary cytoplasmic components are degraded by lysosomes which can result in accumulation of defective proteins, and in a process known as proteotoxicity (Tarone, 2014; Wang and Wang, 2015).

CR is also associated with oxidative stress due to an increase in the production of reactive species with the reduction of antioxidant defenses; this phenomenon can lead to several conditions, such as lipid peroxidation, changes in proteins responsible for calcium transit, activation of signaling pathways for hypertrophy, protein oxidation, DNA damage, cellular dysfunction, proliferation of fibroblasts, activation of metalloproteinases, stimulation of apoptosis, among others (Münzel et al., 2015).

Recent studies uncovered miR-22 as an important regulator for CR. miR-22 modulates the expression and function of genes involved in the hypertrophic response, sarcomere reorganization, and metabolic program shift during CR (Huang and Wang, 2014; Liu et al., 2015).

In CR there is evidence of changes in the calcium transport system, such as decrease of the L-channels and ryanodine receptors, as well as a decrease in the activity of calsequestrin and calmodulin kinase, and a reductionin in phospholamban phosphorylation. These alterations result in a decreased of supply of calcium during systole and increased calcium in diastole. These changes in the proteins in calcium transit may contribute to cardiac dysfunction (Luo and Anderson, 2013; Feridooni et al., 2015).

In addition, the collagen content plays a critical role in the maintenance of the architecture and cardiac function. In the CR process, there may be an imbalance between the synthesis and degradation of collagen with several deleterious effects, such as increased myocardial stiffness, diastolic dysfunction, worsening in coronary flow and the occurrence of malignant arrhythmias (Spinale et al., 2013; Deb and Ubil, 2014; Leask, 2015; López et al., 2015).

The present research topic also provides readers with fundamental advancement in our understanding of the regulation of CR. Indeed, in an elegant study, Gibb et al. examined nocturnal/diurnal behavior, treadmill exercise compliance, and systemic as well as cardiac-specific exercise adaptations in two commonly used mouse strains C57BL/6J, and FVB/NJ mice. FVB/NJ strain is a useful and robust mouse model for examining cardiac adaptations to treadmill exercise and that treadmill training during daytime hours does not negatively affect exercise compliance or capacity. Finally, they provide new insights to demonstrate that FVB/NJ mice are a suitable and robust model for understanding the mechanisms underlying cardiac adaptations to exercise (Gibb et al.).

In other article, Ruiz-Hurtado et al. investigated whether mild (20% food intake reduction) and short-term (2-weeks) caloric restriction prevented the obese cardiomyopathy phenotype and improved the metabolic profile of young (14 weeks of age) genetically obese Zucker fa/fa rats. The results suggest that with mild and short-term caloric restriction prevented an obesity-induced cardiomyopathy phenotype in young obese fa/fa rats independently of the cardiac metabolic profile.

Bai et al. contributed a very interesting review paper focused on the mechanisms underlying the emergence of post-acidosis arrhythmia at the tissue level, altered source-sink interactions and electrical heterogeneity due to acidosis-induced cellular electrophysiological alterations which may increase susceptibility to post-acidosis ventricular arrhythmias.

Due to the diversity and high quality of the articles compiled here, we hope that this can offer new ideas and research projects for new advances in the field.

## Author contributions

LR: Substantial contributions to the conception or design of the work; or the acquisition, analysis, or interpretation of data for the work; Drafting the work or revising it critically for important intellectual content; Final approval of the version to be published. AP: Substantial contributions to the conception or design of the work; or the acquisition, analysis, or interpretation of data for the work; Drafting the work or revising it critically for important intellectual content; Final approval of the version to be published.

### Conflict of interest statement

The authors declare that the research was conducted in the absence of any commercial or financial relationships that could be construed as a potential conflict of interest.
